# P-1008. Congenital Cytomegalovirus Infection Screening in the Newborn Nursery: Areas for Improvement in the Absence of Standardized Guidelines

**DOI:** 10.1093/ofid/ofae631.1198

**Published:** 2025-01-29

**Authors:** Tanya Abraham, Ankita P Desai, Nori M Minich

**Affiliations:** University of Maryland Medical Center, Baltimore, Maryland; University Hospitals Rainbow Babies and Children's Hospitals, Cleveland, Ohio; Case Western Reserve University School of Medicine, Cleveland, Ohio

## Abstract

**Background:**

Cytomegalovirus (CMV) is the most common viral cause of congenital defects in the U.S. Early identification of congenital CMV (cCMV) infection allows for optimal antiviral treatment which reduces progression of sensorineural hearing loss and improves neurodevelopmental outcomes. There is varying guidance from expert consensus groups ranging from universal screening to selective screening based on clinical features. With conflicting data, many hospitals have no standardized protocol for cCMV screening. This study aims to detail clinical characteristics and outcomes of infants with cCMV in a tertiary medical center prior to implementation of standardized guidelines.

**Methods:**

This was a retrospective case study of newborns who screened positive for CMV between June 2017-July 2022 at Rainbow Babies & Children’s Hospital in Cleveland, Ohio. 1900 newborns less than 21 days old were tested for CMV during this period, and newborns who screened positive for CMV were included (n=18). Clinical characteristics of the patients were described using median and interquartile range for continuous variables and frequency and percentages for categorical variables.

**Results:**

The prevalence rate of cCMV at our center is 9.5 per 1000 live births. The most common associated clinical features were small for gestational age (SGA; n=12), microcephaly (n=9), abnormal neuroimaging findings (n=6), and failed newborn hearing screen (n=4). Two patients had isolated SGA, and neither received treatment nor had clinical sequelae. Saliva PCR tests were used for screening with no confirmatory urine testing. 11 patients received oral valganciclovir with documented side effects of transient neutropenia and transaminitis. 33% of newborns had no infectious disease or audiology follow-up.

**Conclusion:**

Targeted screening practices for cCMV infection in our institution varies between providers. Our center has a prevalence rate slightly above the national average; however, the yield of screening isolated SGA is shown to be low. This study highlights gaps in current cCMV screening including lack of confirmatory urine CMV testing, and infants lost to follow up. System-level interventions including creation of standardized guidelines and provider education are necessary to improve follow-up and outcomes.

**Disclosures:**

**All Authors**: No reported disclosures

